# Enhancing Nutrient Recovery and Bioactive Compound Extraction from Spirulina through Supercritical Fluid Extraction: Implications for SH-SY5Y Cell Viability

**DOI:** 10.3390/foods12132509

**Published:** 2023-06-28

**Authors:** Francisco J. Martí-Quijal, Noelia Pallarés, Katarzyna Dawidowicz, María-José Ruiz, Francisco J. Barba

**Affiliations:** 1 Research Group in Innovative Technologies for Sustainable Food (ALISOST), Nutrition, Food Science and Toxicology Department, Faculty of Pharmacy, Universitat de València, Avda. Vicent Andrés Estellés, s/n, 46100 Burjassot, València, Spain; francisco.j.marti@uv.es (F.J.M.-Q.); dawidowicz.ka@wp.pl (K.D.); 2 Research Group in Alternative Methods for Determining Toxics Effects and Risk Assessment of Contaminants and Mixtures (RiskTox), Nutrition, Food Science and Toxicology Department, Faculty of Pharmacy, Universitat de València, Avda. Vicent Andrés Estellés, s/n, 46100 Burjassot, València, Spain; m.jose.ruiz@uv.es

**Keywords:** supercritical fluid extraction, microalgae, minerals, heavy metals, polyphenols, fatty acids, cell viability

## Abstract

This work explores the efficiency of supercritical fluid extraction (SFE) to recover minerals, pigments, and antioxidant compounds from the spirulina microalgae. Moreover, the fatty acids and phenolic profiles of the extracts obtained were also investigated, and the effect of the extracts on SH-SY5Y cell viability was tested. The extraction of phycocyanin was improved by SFE compared to conventional extraction, from 2.838 ± 0.081 mg/g dry matter (DM) (control) to 6.438 ± 0.411 mg/g DM (SFE). SFE treatment also improved chlorophyll a and carotenoid recoveries increasing from 5.612 ± 0.547 to 8.645 ± 0.857 mg/g DM and from 0.447 ± 0.096 to 0.651 ± 0.120 mg/g DM, respectively. Regarding minerals, the SFE improved Mg recovery with 77% more than the control extraction. Moreover, palmitoleic, stearic, γ-linolenic, eicosadienoic and eicosatrienoic acids recovery was improved by SFE. Phenolic profiles were identified via triple-TOF-LC-MS-MS. Considering heavy metals, a higher rate of Pb extraction was observed for the SFE extract, while no significant differences were observed for Hg between both extractions. Finally, SFE extract improved cell viability compared to the control extract. Thus, SFE constitutes an interesting tool to sustainably extract high-added-value compounds; however, potential contaminants such as Pb need to be controlled in the resulting extracts.

## 1. Introduction

In recent years, the interest in the consumption of fresh products rich in nutrients and bioactive compounds has increased due to their potential use and benefits in the medical and pharmaceutical industries [1]. In this context, microalgae contain polysaccharides and phycobiliproteins that have interesting properties to improve the rheological and nutritional properties of food matrices, while polyphenols and xanthophylls are associated with interesting bioactivities, including antioxidant or cytotoxic effects [2]. Due to the interest in algae as a source of food supplements, new strategies should be pursued to valorize the functional constituents extracted from them for use in the food, feed, and cosmetic industries, as well as for the production of pigments and additives. In addition, the extraction of bioactive compounds from microalgae is interesting because it presents an opportunity for sustainable processing [3].

Spirulina is a cyanobacteria that belongs to the blue microalgae and is widely dis-tributed in South America. Due to its composition and the health benefits associated with its consumption, this microalga has an interesting potential to become an important food in the future and to be used as an ingredient in the development of functional foods [4]. It contains a high concentration of nutrients such as proteins, vitamins, minerals, and fatty acids, especially omega-3 and omega-6 fatty acids, which contribute to basic human nu-trition [5]. It is also rich in chlorophylls (Chls), carotenoids (Car), phycocyanins (PC), and phenolic compounds that can be used as colorants and natural antioxidants [6,7].

Conventional extraction techniques such as percolation, maceration, countercurrent extraction, and Soxhlet require the use of toxic organic solvents such as chloroform, methanol, acetone, and diethyl ether in large quantities and for long periods of time. Moreover, they could affect the biological activity of the extracted molecules [8,9]. The use of environmentally friendly technologies such as supercritical fluid extraction (SFE), pulsed electric fields, high-voltage electric discharges, high-pressure homogenization, ultrasound, and microwave-assisted extraction have overcome many limitations of conventional extraction methods. With these technologies, valuable compounds can be extracted from microalgae without using toxic solvents, thus minimizing the environmental impact [10,11].

Supercritical fluid extraction (SFE) is a cost-effective extraction method that follows the principles of green chemistry. It enables the recovery of extracts with high extraction efficiency and selectivity by adjusting the parameters of temperature, pressure, and flow rate. SFE shows promise in applications in food processing and in the extraction of metals in the form of complexes and functional ingredients from natural sources. It can also be an interesting tool for the decontamination of hazardous substances [12]. The main limitation of this technology is the chemical behavior of CO_2_ under supercritical conditions. In this sense, the addition of a suitable polar co-solvent changes the polarity of SFE-CO_2_ and increases the solubility of nutrients and bioactive compounds [13]. This technique has been used by several authors to separate and purify nutrients and bioactive compounds from spirulina, with good results [14,15,16].

Finally, extracts obtained from microalgae are often rich in a variety of nutrients, vitamins, minerals, antioxidants, and other biologically active compounds that can promote cell growth, survival, and overall viability. By creating a nutrient-rich environment, microalgae extracts have the potential to improve cell viability and stimulate cell proliferation [17]. Moreover, these extracts usually contain potent antioxidants such as carotenoids, phycobiliproteins, and tocopherols, which effectively protect cells from oxidative stress [18]. In addition, certain microalgae extracts possess anti-inflammatory properties due to bioactive compounds such as omega-3 fatty acids, PC, and polysaccharides [19]. Considering these factors, it is interesting to study the effects of these extracts on cell viability to determine if they have beneficial effects on cellular functions.

The aim of this work is to investigate the recovery of total phenolic compounds (TPC), pigments, PC, and antioxidant active compounds from spirulina algae using SFE. The study compares the results obtained with SFE to those obtained with conventional stirred extraction (control). Additionally, the fatty acid profile of both SFE and conventional extracts is analyzed. The research also explores the potential of SFE in mineral recovery and monitors the presence of heavy metals in the extracted compounds. The primary objective is to provide an overview of the different compounds of nutritional interest extracted by each method. Furthermore, the study examines the effects of these extracts on cell viability over a 24-h exposure period to determine any potential beneficial effects on cells. Notably, the novelty of this research lies in the fact that there are currently no existing studies in the literature that evaluate the effects of spirulina extracts obtained through SFE on cell viability.

## 2. Materials and Methods

### 2.1. Chemicals

Sodium carbonate (Na_2_CO_3_), sodium hydrogen phosphate (Na_2_HPO_4_), and potassium dihydrogen phosphate (KH_2_PO_4_) were acquired from VWR (Saint-Prix, France). Nitric acid (HNO_3_) was obtained from Panreac (Barcelona, Spain). Methanol, absolute ethanol (EtOH) and hexane were purchased from VWR Chemicals (Rosny-sous-Bois, France). CO_2_ was obtained from Carburos Metálicos (Massalfassar, Valencia, Spain). EtOH 96° was obtained from Guinama (La Pobla de Vallbona, Valencia, España). Folin–Ciocalteu reagent, potassium persulfate (K_2_S_2_O_8_), gallic acid, ABTS (2,2-Azino-Bis-3-eThylbenzothiazoline-6-Sulphonic Acid), fluorescein, 2,2-Azobis(2-amidinopropane) dihydrochloride (AAPH), and trolox (6-hydroxy-2,5,7,8-tetramethylchroman-2-carboxylic acid) were purchased from Sigma Aldrich (Steinheim, Baden-Württemberg, Germany).

### 2.2. Samples

The spirulina samples were obtained from Hainan Island (China). The microalga was grown in open flow-through ponds. No artificial light was used, and shade nets partially covered the culture ponds, allowing control of pigment production. At the time of harvest, all samples were dried with dry air at 60 °C. 

### 2.3. Extraction Process

#### 2.3.1. Shaking Extraction

A shaking extraction process was performed following the protocol described by Sansone et al. [20]. Briefly, 5 g of microalgae was suspended in 50 mL of pure EtOH in the dark shaking at 500 rpm for 30 min. Then, the mixture was separated via centrifugation at 4000 rpm for 10 min, and the liquid phase was transferred to a clean tube. The pellet was resuspended in 50 mL of EtOH and extracted a second time. After repeating the separation process via centrifugation, both extracts were pooled.

#### 2.3.2. Supercritical Fluid Extraction (SFE)

The supercritical fluid extraction (SFE) was conducted using a JASCO system (Tokyo, Japan). The system consisted of various components: an isocratic CO_2_ pump (PU-4387) capable of a flow rate between 5 and 40 mL/min, adjustable in increments of 0.01 mL/min, with a maximum pressure of 50 MPa and a pulse extraction system; a cooling circuit (JULABO FL 1201) to cool the pump, offering a temperature range of −20 to 40 °C, a flow rate of up to 23 L/min, and a cooling power of 1.2 kW at 20 °C; an isocratic pump for organic modifiers (PU-4086, HPLC) with a flow rate range of 0.001–10 mL/min, adjustable in 0.01 mL/min increments, and a peak pressure of 70 MPa; a thermostatic oven for glass reactions (CO-4065) with a temperature range of 4 to 90 °C; a thermostatic system employing Peltier and temperature transfer via air flow; a pressure regulator (BP-4340); and a 5 mL glass vessel for SFE (803,559–5 mL). The extraction process lasted for 1 h, following a modified version of the method described by Mendes et al. [21]. Specifically, the extraction vessel was filled with 5 g of dried spirulina, and the procedure used a temperature of 50 °C, a pressure of 25 MPa, and a CO_2_:EtOH ratio of 90:10, with a flow rate of 16 mL/min.

### 2.4. Measure of Antioxidant Capacity, Pigments and Polyphenols

#### 2.4.1. Total Antioxidant Capacity

The measure of total antioxidant capacity was carried out by two different methods: Trolox equivalent antioxidant capacity (TEAC) and oxygen radical antioxidant capacity (ORAC) tests. These assays were carried out following the protocol described by al Khawli et al. [22]. The results were expressed as µmol Trolox equivalents (TEs)/g dry matter (DM). The experiments were performed in triplicate.

#### 2.4.2. Pigments and Total Phenolic Content

The content of chlorophyll a (Chla) and total carotenoids (Car) was calculated based on the procedure described by Lichtenthaler and Wellburn [23] with the modifications of Lima et al. [24] for spirulina microalgae. The different equations used are:

Chla = 15.65 × A_666_ − 0.766 × A_644_ (1)

Car = (1000 × A_470_ − 2.86 × Chla)/245 (2)

The total phenolic content (TPC) analysis was performed following the method described by Martí-Quijal et al. [25]. Briefly, 3 mL of Na_2_CO_3_ were added to a test tube, then 100 μL of standard or sample extract (appropriately diluted) and finally, 100 μL of Folin–Ciocalteu reagent were also added. Then, the samples were incubated for 60 min in darkness at room temperature measured at a wavelength of 765 nm.

The concentration of PC was determined following the protocol described by Patil et al. [26], measuring the absorbance at 620 nm. The results were expressed as mg/g DM.

### 2.5. Evaluation of Minerals and Heavy Metals Content

The minerals analyzed were Mg, P, Ca, Fe, Zn and Se, as they are the more interesting minerals from a nutritional point of view, being essential for several biological processes [27]. On the other hand, the heavy metals studied were As, Cd, Hg, and Pb, as they are the main ones in marine resources and they are also among the minerals with the greatest adverse effects [28]. All of them were identified and quantified via ICP-MS, following the method previously used by de la Fuente et al. [29] for heavy metals. To sum up, 1 mL of the obtained extracts diluted at 1 mg/mL was digested with 250 µL of H_2_O_2_ (35% *v*/*v*) and 1 mL of HNO_3_ (69% *v*/*v*) in a microwave system (MARS, CEM, Vertex, Spain) (800 W and 180 °C for 15 min). Then, the samples were filtered and diluted to an appropriate volume using ultrapure water. Finally, the content of minerals (Mg, P, Ca, Fe, Zn and Se) and heavy metals (As, Hg, Cd, and Pb) was evaluated using an inductively coupled plasma spectrometer mass detector (ICP-MS, Agilent model 7900). For the quantification, a standard calibration curve was used. Results are expressed as µg/g DM of three independent experiments.

### 2.6. Fatty Acids Profile Analysis

The determination of lipid profile was carried out via gas chromatography coupled to flame ionization detector (GC-FID). Briefly, 1 mL of the extract resuspended at 20 mg/mL was mixed with 1 mL of KOH 2 N in methanol and 1 mL of hexane. After mixing them for 2 min, the hexane phase was taken and 2 µL were injected in a PerkinElmer Gas Chromatograph Clarus^®^ 590. A standard FAME Mix (Supelco 37 Component FAME Mix, Sigma-Aldrich, Laramie, WY, USA) was used to identify the fatty acids.

The GC column SP-2560^®^, CPWAX 52CB silica capillary WCOT column with a dimension of 30 m of length, 0.25 mm internal diameter with a 0.25 μm thick cover (SUPELCO) SP-2560 Capillary GC Column was operated in split mode. The oven temperature program was established as 180 °C for 5 min, increased to 210 °C at a rate of 4 °C/min, and finally held at 250 °C for 20 min, increasing at a rate of 20 °C/min. Nitrogen was used as a carrier gas at a flow rate of 20 psi. The results were expressed as mg/g DM.

### 2.7. Phenolic Profile

The phenolic profile was analyzed using triple-TOF-LC-MS-MS characterization, following the method described by Zhou et al. [30]. In this study, samples were diluted at 10 mg/mL in methanol and filtered through a 0.22 µm filter. Then, they were directly injected in the equipment. The TripleTOF™ 5600 (ABSCIEX) LC/MS/MS system, coupled with the Agilent 1260 Infinity (Agilent, Waldbronn, Germany), was employed for phenolic compound identification. Chromatographic separation was achieved using a Waters UPLC C18 column (1.7 µm, 2.1 × 50 mm) Acquity UPLC BEH.C18 obtained from Waters (Cerdanyola del Vallès, Spain). The mobile phase consisted of water (0.1% formic acid, A) and methanol (0.1% formic acid, B). The elution gradient of the mobile phase was as follows: 90% (A) and 10% (B) from 0 to 13 min, 100% (B) from 13 to 15 min, and 90% (A) and 10% (B) from 15.1 to 22 min. The flow rate was set at 0.4 mL/min, and an injection volume of 5 μL was used. Mass spectrometric data were acquired in the mass range of 80–1200 *m*/*z*. Prior to sample analysis, calibration of the equipment was performed using an external calibration system. The MS operated using an information-dependent acquisition (IDA) method, employing the survey scan type (TOF-MS) and the dependent scan type (product ion) with a collision energy of −50 V. The MS parameters were as follows: ion spray voltage of −4500 V, dust removal potential of 90 V, collision energy of −50 V, temperature of 400 °C with a curtain gas of 25 psi, ion source gas 1 at 50 psi, and ion source gas 2 at 50 psi. The IDA MS/MS was performed based on the following criteria: ions exceeding 100 CPS, ion tolerance of 50 MDa, collision energy set at 25 V, and dynamic background subtraction enabled.

### 2.8. Cell Cultures and Cell Viability Assay

#### 2.8.1. Cell Culture

For cell viability analysis, SH-SY5Y cells (ATCC-CRL-2266, ATCC, New York, NY, USA), a type of human neuroblastoma cells, were cultured in DMEM Ham’s-F12 medium supplemented with 10% fetal bovine serum (FBS), 100 U/mL penicillin, and 100 mg/mL streptomycin. The cells were incubated under specific conditions: pH 7.4, 5% CO_2_ at 37 °C, and 95% air atmosphere with constant humidity. The culture medium was refreshed every 2–3 days. To test the effects of various concentrations of spirulina extracts, the dry matter obtained after the extraction was resuspended in DMSO, and the solutions were added to the culture medium, ensuring that the final DMSO concentration did not exceed 1% (*v*/*v*). Control groups containing the same amount of solvent were included in each experiment.

#### 2.8.2. Cell Viability Assay

To assess cell viability, the MTT assay was conducted as described by Zingales et al. [31]. In brief, 30,000 cells/well were seeded in 96-well plates and allowed to grow for 48 h until reaching 80% confluence. Subsequently, the cells were exposed to different concentrations of spirulina extracts (ranging from 125 to 2000 μg/mL) for 24 h. After the incubation period, the culture medium containing the spirulina extract was replaced with fresh medium containing 50 μL of MTT salt (5 mg/mL in PBS). Following 3 h of incubation at 37 °C in darkness, the resulting formazan crystals were dissolved in DMSO. An automatic plate reader (MultiSkanEX, Labsystem, Helsinki, Finland) was used to measure absorbance at 540 nm. Cell viability was expressed as a percentage relative to the solvent control (1% DMSO).

### 2.9. Statistical Analysis

The statistical analysis was performed using GraphPad Prism 9. Student’s *t* test was used to compare SFE and control samples. A *p* value of <0.05 was considered significant. All the experiments were carried out in triplicate. The results are presented as mean ± SD.

## 3. Results and Discussion

### 3.1. Pigments and Total Phenolic Content

Chlorophyll, Car, and polyphenols are bioactive compounds found in vegetables and algae that have been extensively studied for their antioxidant, anti-inflammatory and an-ticancer properties [32,33]. PC, a protein extracted from spirulina, has also been shown to have various biological functions, including antioxidant and anti-inflammatory effects, inhibition of bacterial growth, and protection against liver and kidney damage, making it a promising natural molecule for various applications [32,34]. PC and other phycobiliproteins, along with Chls and Car, are considered essential algal pigments [2].

**Figure 1:**
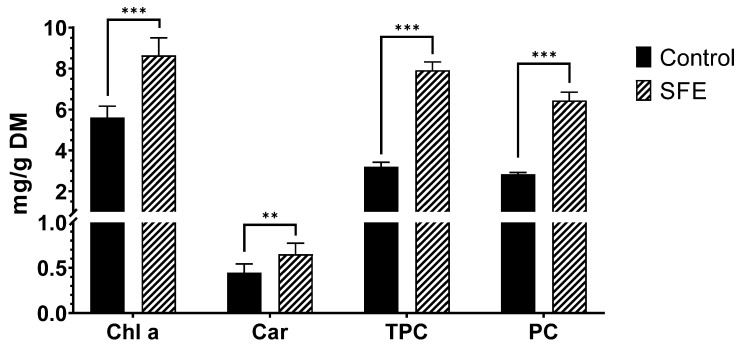
Chlorophyll a (Chl a), total carotenoids (Car), total phenolic content (TPC), and phycocyanin (PC) values comparing SFE vs. control extraction. Results are expressed as mean ± SD. ** = *p <* 0.01. *** = *p <* 0.001.

The Chla, total Car, TPC, and PC values in both the control and SFE extracts are shown in Figure 1.

As shown in Figure 1, SFE treatment significantly improved pigment extraction compared with the control, from 5.612 ± 0.547 to 8.645 ± 0.857 mg/g DM for Chl a, and from 0.447 ± 0.096 to 0.651 ± 0.120 mg/g DM for Car. These results are in close agreement with the published literature. In the same vein, Tong et al. [35] described an increase in Chl a from 2.73 ± 0.05 to 6.84 ± 0.18 mg/g using SFE compared to the control for 120 min. These results show a 2.5-fold increase in Chl a extraction, which is higher than the 1.53-fold obtained in our study. However, this fact can be explained by considering the extraction time, which is 2 h in Tong et al. whereas we chose an extraction time of 1 h in our study, based on the optimal conditions found in the available literature. In addition, other parameters such as pressure may also affect the results, as Tong et al. performed the extraction at 48.7 MPa, while only 25 MPa were used in our study.

The low yield in Car extraction may be attributed to pressures lower than 30 MPa, as suggested by Marzorati et al. [36]. They found that pressures below 30 MPa were inadequate to obtain Car-enriched extracts. In addition, a higher yield of Car can be achieved when a modifier phase is not used during extraction. However, in our study, we utilized 10% EtOH as a co-solvent. The same authors reported total Car value of 3.5 ± 0.2 mg/g DM after SFE under 30 MPa and 45 °C at a flow rate of 15 mL/min and without co-solvent. However, other authors also obtained lower values for carotenoid from spirulina via SFE. For instance, Esquivel-Hernández et al. [37] obtained 0.283 mg of carotenoids/g DM using SFE (CO_2_) with a flow rate of 15 g/min of CO_2_ for 50 min and 26.70% EtOH 96%/water (*v*/*v*) as a co-solvent under 15 MPa and 60 °C. Despite the similarity in flow rate and extraction time, the pressure used by these authors was lower, and the percentage of EtOH was higher. These differences may account for our higher value of Car. 

SFE extraction also increased the recovery of phenolic compounds, resulting in an increase from 3.202 ± 0.547 mg/g DM using conventional extraction to 7.917 ± 0.406 mg/g DM with SFE. These findings align with those reported by Mallikarjun et al. [15], who obtained a value of 340 mg/100 g DW (=3.4 mg/g DM) using SFE at 40 °C, a pressure of 12 MPa, and CO_2_ flow rate of 1.2 Kg/h. Additionally, the results obtained through SFE correspond well with the values reported by Dejsungkranont et al. [38], who recorded TPC values ranging from 6.44 to 11.40 mg gallic acid equivalents (GAE)/g DM. The highest values were achieved using 40 °C, 31.03 MPa and 90 min of extraction (11.10 mg GAE/g DM), and 60 °C, 24.13 MPa, and 90 min (11.40 mg GAE/g DM). Notably, the extraction time in both studies was 90 min, which is 30 min longer than what we used in our study. This, coupled with the use of a higher pressure or temperature, likely accounts for the higher values obtained. It is evident that the duration and conditions of the extraction process significantly impact the outcome of the experiment. 

Regarding the value obtained for PC, a significant increase in the extraction yield of this protein was observed after using SFE, compared to the control extraction under stirring, improving from 2.838 ± 0.081 mg/g DM (control) to 6.438 ± 0.411 mg/g DM (SFE), which means an increase of 126.8%.

Deniz el al. [39] used SFE to optimize the PC extraction from spirulina, studying the influence of temperature, pressure, time and percentage of co-solvent (EtOH) and comparing to a control. These authors reported that the maximum PC value with the highest purity was obtained at 60 °C, 25 MPa, 45 min and 10% of EtOH as co-solvent. These values are quite similar to the ones used in the present study (50 °C, 25 MPa, 60 min and 10 % of EtOH as co-solvent). In addition, Deniz et al. also found a higher extraction of PC when used SFE compared to a control, being 13% higher.

Pinto et al. [40] studied the extraction of PC and other lipids from spirulina comparing SFE (only CO_2_), SFE (with 10% EtOH as co-solvent), and pure EtOH at high pressure. The results were in agreement with the obtained in the present study, as they obtained a higher PC extraction when using SFE with 10% EtOH as co-solvent compared with pure EtOH. The PC extraction improved from 0.6  ±  0.2 up to 0.9 ± 0.3 (wt%), which means an increase of 150%.

### 3.2. Antioxidant Capacity

**Figure 2:**
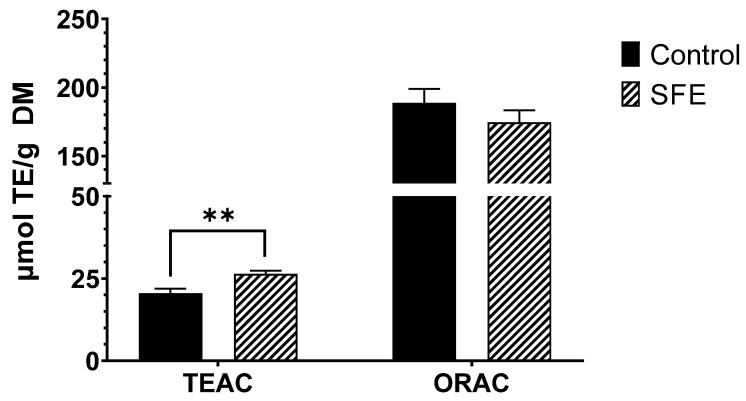
TEAC and ORAC antioxidant assays, comparing SFE vs. control. Results are expressed as mean ± SD. ** = *p <* 0.01.

The results obtained regarding the antioxidant capacity (Figure 2) demonstrate an increase in the measured antioxidant capacity via the TEAC assay. The value rose from 20.6 ± 1.35 µmol TE/g DM to 26.4 ± 0.99 µmol TE/g DM after the SFE extraction. This increase in TEAC value could be related to the improved extraction of antioxidant compounds, particularly polyphenols, previously described in the study. The TEAC assay has a very good correlation with the concentration of polyphenols in the sample, which effectively represents their antioxidant activity [41].

On the other hand, the ORAC assay did not show significant differences between the control (189 ± 10.2 µmol TE/g DM) and the SFE (175 ± 8.60 µmol TE/g DM) samples.

The results obtained in the present study for the TEAC assay were slightly lower compared to those obtained by Dejsungkranont et al. [38]. They achieved antioxidant activity values, measured via the DPPH (2,2-Diphenyl-1-picrylhydrazyl) assay, in the range of 44.18 to 118 µmol TE/g DM after using SFE in samples of *Spirulina maxima*. However, this can be explained by the higher polyphenol values obtained by those authors, as discussed earlier, which correspond to a higher antioxidant capacity than what was found in our study.

### 3.3. Mineral Content

Macrominerals are essential for a good health. Magnesium is a vital cofactor in over 300 enzyme systems and is essential for energy production, cell membrane transport, nerve impulse conduction, muscle contraction, and heart rhythm. Low Mg levels are associated with various chronic health conditions such as migraines, osteoporosis, Alzheimer’s disease, asthma, hypertension and insulin resistance [42]. Calcium and phosphorus are essential macrominerals for neuromuscular function and skeletal mineralization. Calcium is abundant in bones and teeth and plays a critical role in blood vessel contraction, muscle tone, and nerve transmission. Phosphorus is primarily found in mineralized bone and is necessary for DNA, RNA, and ATP synthesis. Parathyroid hormone (PTH), vitamin D and, fibroblast growth factor 23 (FGF23) control the levels of both minerals [42].

On the other hand, microminerals (e.g.,: Fe, Zn, Se…) are also relevant for several functions in human body. In this sense, selenium’s nutritional benefits are achieved through 25 selenoproteins that contain selenocysteine. During low selenium supply, some selenoproteins are prioritized, including glutathione peroxidase. Selenoproteins play a vital role in human health. For example, Se supplementation can stimulate the immune system, including enhancing T cell proliferation and natural killer cell activity [43]. Zinc is a vital trace element in the human body that acts as a signaling factor and regulates chronic inflammation. It also aids in the synthesis of antioxidant enzymes and catalyzes enzymes involved in metabolism. Furthermore, Zn plays a critical role in insulin synthesis and storage, making it important for metabolic disorders such as type-2 diabetes mellitus and atherosclerosis [44]. Finally, iron is crucial for various metabolic processes such as oxygen transport, DNA synthesis, and electron transport in living organisms. Anemia or functional impairments can result from Fe deficiency. Even mild or moderate forms of Fe deficiency anemia can lead to cognitive, immune, and work capacity deficits [45].

**Figure 3:**
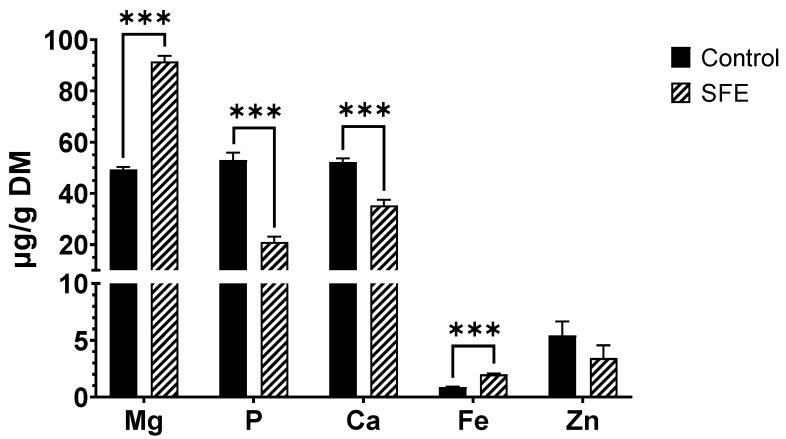
Content of Mg, P, Ca, Fe, and Zn in both SFE and control extracts. Se was not detected. Results are expressed as mean ± SD. *** = *p <* 0.001.

Then, concerning the minerals, Mg, P, Ca, Fe, Zn and Se, the SFE recovered 77% more Mg than the conventional extraction, increasing from 49.33 ± 1.00 up to 91.50 ± 2.25 µg/g DM. In addition, Fe extraction was also improved through SFE, reaching 2.00 ± 0.08 µg/g DM for SFE extract while only 0.87 ± 0.04 µg/g DM for the conventional one. However, the conventional extraction obtained a higher yield for the recovery of P (52.98 ± 2.86 vs. 20.93 ± 2.25 µg/g DM) and Ca (52.27 ± 1.43 vs. 35.25 ± 2.25 µg/g DM) (Figure 3). This can be explained due to the lower solubility of minerals in non-polar solvents, like supercritical CO_2_ [46]. Nevertheless, the ability of SFE to recover more Mg from spirulina compared to EtOH extraction could be attributed to the fact that Mg is more soluble in nonpolar solvents than Ca and P. Lastly, no significant differences were observed for Zn, while Se was not detected in any of the studied samples. 

In this sense, Michalak et al. [46] also obtained a better yield recovering Mg (406 ± 61 mg/L from 4070 ± 810 mg/Kg DM) than Ca (1060 ± 210 mg/L from 14,400 ± 2900 mg/Kg DM) and P (43 ± 6 mg/L from 1520 ± 300 mg/Kg DM) after the extraction of supercritical fluids from Baltic seaweeds.

### 3.4. Heavy Metals Content

**Figure 4:**
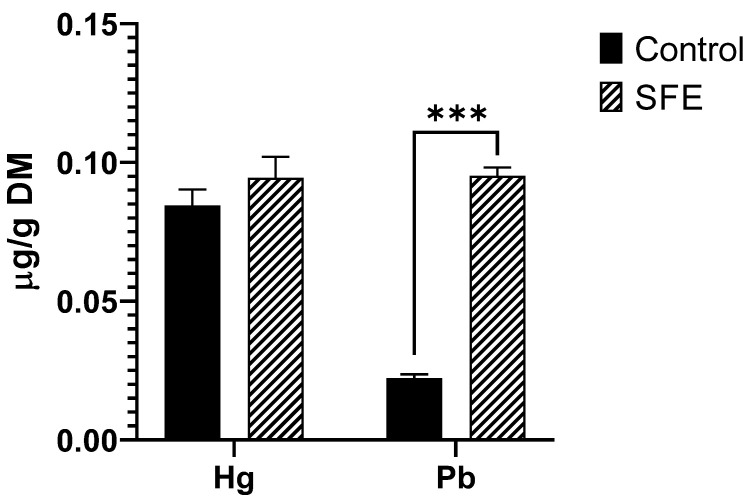
Content of heavy metals Hg and Pb in both SFE and control extracts. As and Cd were not detected. Results are expressed as mean ± SD. *** = *p <* 0.001.

For heavy metals (As, Cd, Hg and Pb), a higher Pb extraction was observed for the SFE extract (0.0953 ± 0.0030 µg/g DM) compared to control (0.0222 ± 0.0014 µg/g DM), while for Hg there were no significant differences between both extractions (Figure 4). Neither As nor Cd were found in any of the samples.

Commission Regulation (EC) No 1881/2006 [47] established maximum levels for some heavy metals (such as As, Cd, Pb, and Hg) in various food products, but did not establish any maximum levels for algae and halophilic plants, except for food supplements composed exclusively or mainly of seaweed. Recently, in the case of Hg, Regulation (EC) No 2018/464 [48] set a maximum residue limit (MRL) of 0.01 mg/Kg for algae and prokaryotic organisms. However, these levels are regulated based on wet weight, while our samples are measured based on dry weight, resulting in a more concentrated result than the value on a wet weight basis.

To the best of our knowledge, no studies are available in the literature about heavy metals contents in spirulina SFE extracts, so the contents obtained in this study have been compared with the information available in raw spirulina. In this sense, Al-Harbi [49] studied the occurrence of Pb, As and Cd in 25 spirulina products commercialized for human consumption. The concentrations reported by these authors were Cd (0.003–0.069), As (0.006–0.578), Pb (0.100–1.206) expressed in mg/Kg dry weight, being Pb levels similar to those observed in the present study after SFE. Contrary to the present study, Hsu et al., [50] reported As levels up to 2 µg/g in spirulina food samples, while Pb was detected in one sample at 15 µg/g. Pb levels were higher than those determine in the present study. Moreover, these authors detected Hg at lower contents (<0.03 µg/g).

### 3.5. Fatty Acids Profile

Fatty acids are vital for human health, serving as building blocks for cell membranes and precursors for signaling molecules. Palmitoleic acid, a monounsaturated fatty acid, has been linked to lower inflammation levels and improved insulin sensitivity [51]. Linoleic acid is also associated with lower type 2 diabetes risk and potential insulin sensitivity improvement [52]. Stearic acid, a saturated fatty acid, has a neutral effect on cholesterol levels and may even lower cholesterol when replacing other saturated fats in the diet [53]. Additionally, polyunsaturated fatty acids like γ-linolenic acid, eicosadienoic acid, and eicosatrienoic acid have anti-inflammatory properties and may reduce the risk of chronic diseases such as arthritis, diabetes, and heart disease [54].

**Figure 5:**
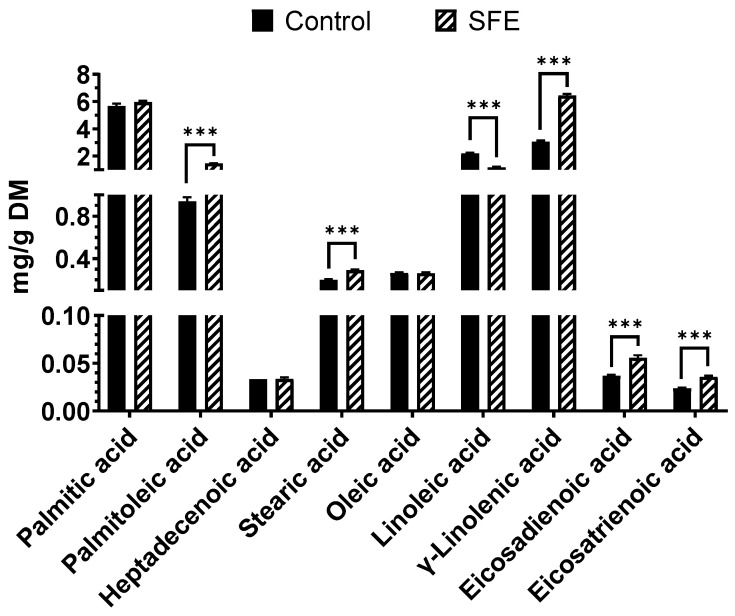
Fatty acids profile in both SFE and control extracts. Results are expressed as mean ± SD. *** = *p <* 0.001.

Figure 5 illustrates the fatty acid profile of both the control and SFE extractions. The SFE method enhanced the recovery of several fatty acids, including palmitoleic, stearic, γ-linolenic, eicosadienoic, and eicosatrienoic acids. The most significant increase was observed in γ-linolenic acid, which rose by 110% from 3.056 ± 0.100 mg/g DM in the control to 6.436 ± 0.120 mg/g DM in the SFE sample. Palmitoleic and stearic acids also experienced a substantial increase of 55.84% and 45.05%, respectively, from their control values of 0.941 ± 0.038 and 0.200 ± 0.007 mg/g DM. Similarly, eicosadienoic and eicosatrienoic acid increased by around 50% following SFE. Specifically, eicosadienoic acid increased from 0.037 ± 0.001 to 0.056 ± 0.002 mg/g DM, while eicosatrienoic acid increased from 0.024 ± 0.001 to 0.036 ± 0.001 mg/g DM.

However, there was a reduction in linoleic acid content, with the control extraction having a higher concentration (2.200 ± 0.060 mg/g DM) than the SFE extraction (1.180 ± 0.042 mg/g DM).

On the other hand, Table 1 displays the relative proportion of each fatty acid that was determined in both the control and SFE extracts. As shown in Table 1, the SFE process promoted a slight modification in the proportion of each acid when compared to the control extraction. The most noticeable changes were observed in palmitic acid and linoleic acid, which underwent a reduction of around 6–7%, decreasing from 53.24% to 46.90% for palmitic acid and from 15.60 to 7.02% for linoleic acid. The reduction in the case of linoleic acid was particularly striking, as its relative presence in the extract is reduced by 50%, compared to the initial proportion, after the application of SFE.

Conversely, the most significant increase occurs in γ -linolenic acid, which rises from 20.66% in the control extract to 36.49% in the SFE extract. It is also worth noting that long-chain polyunsaturated acids, such as eicosadienoic acid and eicosatrienoic acid, observing an increase in their proportion in the SFE extract compared to the control, with an increment from 0.25% and 0.17% to 0.33% and 0.21%, respectively. This implies that SFE can enhance the lipid profile in a way that promotes the extraction of unsaturated fatty acids.

Finally, the only fatty acid that does not experience a significant modification regarding its proportion in the obtained extract is heptadecenoic acid, obtaining very similar values in both extracts.

The results obtained in this study are similar to those described in the literature. In this regard, the values are within the same range as those obtained by Esquivel-Hernández et al. [55], with a very similar proportion of fatty acids: palmitic acid (39.38%), γ-linolenic acid (30.27%), linoleic acid (20.63%), palmitoleic acid (7.88%), oleic acid (1.06%), and stearic acid (0.75%). The proportion of these fatty acids also fits with that found by Crampon et al. [56] regarding the composition present in the microalgae. It is described that the major component is palmitic acid, with 63.48%, followed by linoleic acid (13.73%), linolenic acid (9.42%), and palmitoleic acid (8.02%), among others. Therefore, in our case, the extraction of γ-linolenic acid has been notably increased compared to the relative content in the microalgae, obtaining values much higher than 9.42% (20.66% for control and 36.49% for SFE).

Mendiola et al. [14] have also reported the ratio of different fatty acid areas after the application of SFE to 75 g of spirulina with 10% EtOH, 220 bar, and 27 °C. However, these authors detected the presence of lauric acid (3.53–19.2%) and myristic acid (1.38–2.89%), which were not detected in our study. In contrast, the values reported by these authors for unsaturated fatty acids, specifically linoleic acid (0.66–0.92%) and palmitoleic acid (5.80–5.92%), were lower than those obtained in our experiments.

### 3.6. Phenolic Profile

In Table 2, the phenolic compounds identified using triple-TOF-LC-MS-MS with a score equal to or greater than 80% are shown. As can be observed, the use of one treatment or another can influence the type of the recovered compounds. It can be observed that after performing the extraction with SFE, polyphenols such as apigenin 7-O-glucuronide, 3,4-dihydroxyphenylglycol, or acetyl eugenol, among others, were identified in the extracts, which were not present in the control extract. The reason why two or more compounds can appear at the same retention time is because the identification was done tentatively using a library. Therefore, possible compounds have been identified based on their mass, but it cannot be stated which one is definitive due to the absence of patterns and the limitations inherent to the identification method.

The identification of hydroxybenzoic acid, 4-hydroxybenzaldehyde, and protocatechuic acid is in line with what has been described in the literature by Klejdus et al. [57], who identified these compounds after performing an extraction using the combination of solid-phase and supercritical fluid extraction from *Arthrospira platensis*. On the other hand, Zhou et al. [30] detected the presence of phenol in spirulina extracts obtained using pressurized liquid extraction with DMSO as a solvent. The presence of hesperidin in spirulina has also been reported in the literature [58] after extraction with ethanol and methanol. Finally, McCarthy et al. [59] also reported the presence of apigenin in extracts obtained from *Arthrospira platensis*.

### 3.7. Impact on SH-SY5Y Cell Viability

**Figure 6:**
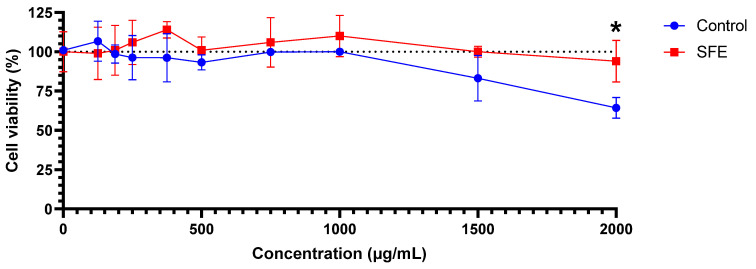
Effect of spirulina extracts (control and SFE) on cell viability in SH-SY5Y cell line. Cell viability was evaluated using MTT assay. Results are expressed as mean ± SD. * = *p <* 0.05 vs. control.

The impact of the SFE spirulina extract on SH-SY5Y cell viability was evaluated and compared to the control extract (Figure 6). To the best of our knowledge, this is the first study in the literature evaluating the cytotoxicity of a SFE spirulina extract, which is crucial for future applications related to human health. The extract concentrations were tested across a wide range: 125, 187.5, 250, 375, 500, 750, 1000, 1500, and 2000 μg/mL. It is worth noting that there was a slight increase in cell viability at the concentration of 375 μg/mL, reaching 114% of cell viability. Moreover, it can be observed that at high extract doses, the cell viability decreased in the control group, while it remained around 100% for the SFE extract. Specifically, at a concentration of 1500 μg/mL, cell viability in the control extract was 83.2%, whereas in the SFE extract, it was 100% compared to the cell viability without the spirulina extract (1% DMSO, used as control for cell viability). The same effect was more pronounced and exhibited significant differences (*p* < 0.05) in the case of the 2000 μg/mL concentration, where the control extraction reduced cell viability to 64.3%, while the SFE extract was similar to control. This was likely due to the higher content of bioactive compounds, as previously described, which help sustain cell viability at high concentrations compared to the control.

Other researchers have examined the cytotoxicity of extracts from this microalga using different methods and solvents. Conversely to our results, Akbarizare et al. [60] found slightly higher results in HepG2 cells. They evaluated the 24 h exposure of aqueous and methanolic spirulina extracts on HepG2 liver cells and human fibroblasts using the MTT assay. In HepG2 cells, they observed an IC_50_ at concentrations of 1700 ± 140 µg/mL (aqueous extract) and 1280 ± 220 µg/mL (methanolic extract). In human fibroblasts, the IC_50_ was reached at concentrations of 2340 ± 60 µg/mL and 2430 ± 40 µg/mL for the aqueous and methanolic extracts, respectively. These concentrations were considerably higher, indicating that our study aligns more closely with the behavior observed in fibroblasts. However, at the range of concentrations tested we did not obtain IC_50_, for either SFE or control extracts.

In contrast, the results differ significantly from those obtained by Nurani et al. [61] for HeLa cells, a type of human cervical cancer cells. They found that the IC_50_ of the ethanolic spirulina extract after 24 h exposure to HeLa cells was 260.4 μg/mL, much lower than our values. However, this difference could be attributed to the intrinsic characteristics of HeLa cells, as in the same study, the authors also tested the extract on human dermal fibroblasts (HDFa cells), obtaining an IC_50_ value of 2065.7 µg/mL, which closely resembles the results of Akbarizare et al. and our results. This confirms that the low IC_50_ value obtained for HeLa cells may be specific to this particular cell line.

In our study, the choice of the cell line was based on the evidence that they represent one of the most common in vitro model used for assessing the toxic effects of several substances [62].

Therefore, it is essential to perform cytotoxicity studies considering the cell line used, the duration of exposure, and conducting a comprehensive characterization of the tested extract.

## 4. Conclusions

In conclusion, this study demonstrates the effectiveness of SFE in efficiently extracting valuable compounds from spirulina microalgae. SFE outperforms conventional extraction methods, resulting in significant improvements in the recovery of phycocyanin, chlorophyll a, carotenoids, and magnesium. It also enhances the extraction of specific fatty acids such as palmitoleic, stearic, γ-linolenic, eicosadienoic, and eicosatrienoic acids. The phenolic profile of the SFE extracts is diverse and promising. However, careful monitoring of potential contaminants, particularly lead (Pb), is necessary. Notably, the SFE extracts show improved cell viability compared to the control extracts when tested on SH-SY5Y cells. Overall, SFE has proven to be a valuable and sustainable method for extracting high-value compounds from spirulina microalgae, with considerations for controlling potential contaminants in the extracts.

## Figures and Tables

**Table 1:** Relative fatty acids concentration both in control and SFE extracts. Results are expressed as mean (%) ± SD.

Fatty Acid	Formula	Relative Concentration (Mean (%) ± SD)	
		Control	SFE
Palmitic acid	C16:0	53.24 ± 0.07	46.90 ± 2.44 *
Palmitoleic acid	C16:1n-7	6.59 ± 0.08	8.28 ± 0.12 ***
Heptadecenoic acid	C17:1n-7	0.23 ± 0.00	0.21 ± 0.01
Stearic acid	C18:0	1.47 ± 0.01	1.79 ± 0.09 **
Oleic acid	C18:1n-9	1.95 ± 0.01	1.63 ± 0.01 ***
Linoleic acid	C18:2n-6	15.60 ± 0.14	7.02 ± 0.28 ***
γ-Linolenic acid	C18:3n-6	20.66 ± 0.01	36.49 ± 1.88 ***
Eicosadienoic acid	C20:2n-6	0.25 ± 0.01	0.33 ± 0.01 ***
Eicosatrienoic acid	C20:3n-6	0.17 ± 0.01	0.21 ± 0.01 **

n.s.: not significant; * = *p <* 0.05; ** = *p* < 0.01; *** = *p <* 0.001 vs. Control.

**Table 2:** Phenolic profile obtained via triple-TOF-LC-MS-MS for the extracts obtained via supercritical fluid extraction (SFE) and its control from spirulina biomass. The extracts were resuspended at 10 mg/mL in methanol. Results are expressed as mean ± SD.

	Compound	Formula	Score	Retention Time (min)
Control	Phenol	C_6_H_6_O	91%	5.52
	Benzoic acid	C_7_H_6_O_2_	90%	6.27
	4-Hydroxybenzaldehyde	C_7_H_6_O_2_	90%	6.27
	2-Hydroxybenzoic acid	C_7_H_6_O_3_	83%	5.51
	3-Hydroxybenzoic acid	C_7_H_6_O_3_	83%	5.51
	4-Hydroxybenzoic acid	C_7_H_6_O_3_	83%	5.51
	Sesamol	C_7_H_6_O_3_	83%	5.51
	Protocatechuic aldehyde	C_7_H_6_O_3_	83%	5.51
	4-Ethylcatechol	C_8_H_10_O_2_	94%	7.80
	4-Vinylsyringol	C_10_H_12_O_3_	87%	8.67
	Hesperidin	C_28_H_34_O_15_	95%	8.46
	Neohesperidin	C_28_H_34_O_15_	95%	8.46
SFE	Phenol	C_6_H_6_O	88%	5.76
	Benzoic acid	C_7_H_6_O_2_	89%	6.53
	4-Hydroxybenzaldehyde	C_7_H_6_O_2_	89%	6.53
	Tyrosol	C_8_H_10_O_2_	87%	8.01
	4-Ethylcatechol	C_8_H_10_O_2_	87%	8.01
	Hydroxytyrosol	C_8_H_10_O_3_	87%	7.69
	3,4-Dihydroxyphenylglycol	C_8_H_10_O_4_	91%	7.62
	Acetyl eugenol	C_12_H_14_O_3_	81%	10.47
	Apigenin 7-O-glucuronide	C_21_H_18_O_11_	86%	6.73

## Data Availability

The data presented in this study are available on request from the corresponding author. The data are not publicly available due to this study belongs to an ongoing research project.
